# F196. DIFFERENTIAL EFFECTS OF MGLU5 RECEPTOR BLOCKADE ON BEHAVIOR, SCHIZOPHRENIA-RELEVANT GENE EXPRESSION AND NEURONAL ACTIVATION PATTERNS FROM DEVELOPMENT TO AGING MICE

**DOI:** 10.1093/schbul/sby017.727

**Published:** 2018-04-01

**Authors:** Dragos Inta, Alessia Luoni, Marco A Riva, Peter Gass

**Affiliations:** 1 Central Institute of Mental Health; 2 University of Milan; 3 Universita’ degli Studi di Milano

## Abstract

**Background:**

The glutamate system is implicated both in schizophrenia and mood disorders. Mice lacking metabotropic mGlu5 receptors (mGluR5 KO) display schizophrenia-like abnormalities. Additionally, mGluR5 antagonists represent promising alternative anxiolytics/antidepressants. However, the underlying age-specific molecular/cellular mechanisms are only partially understood. We aimed at identifying molecular alterations associated with a genetically induced mGluR5 deletion, which results in a schizophrenia-like phenotype. Additionally, we investigated age-specific effects of mGluR5 antagonists on emotional behaviour and c-fos activation.

**Methods:**

For analysis of mRNA and protein levels we performed Real-time RT-PCR and Western blot investigations in the hippocampus and prefrontal/frontal cortex (PFC/FC) of mice with a genetic deletion of the metabotropic glutamate receptor 5 (mGlu5), addressing key components of the GABAergic and glutamatergic systems. Additionally, we used classical behavioral tests for determining anxiety- and depression-like changes triggered by the mGluR5 antagonist 2-Methyl-6-(phenylethynyl)pyridine (MPEP). Finally, we used profiling of c-Fos expression, as marker of neuronal activity, induced by MPEP from postnatal day 16 (P16) to adulthood (P90).

**Results:**

mGlu5 knockout (KO) mice showed a significant reduction of reelin, GAD65, GAD67 and parvalbumin mRNA levels, which is specific for the PFC/FC, and that is paralleled by a significant reduction of protein levels in male KO mice. We also analysed the main NMDA and AMPA receptor subunits, namely GluN1, GluN2A, GluN2B and GluA1, and observed that mGlu5 deletion determined a significant reduction of their mRNA levels, also within the hippocampus, with differences between the two genders. We measured age-specific alterations in emotional behaviour of mGluR5 KO mice, with marked increase of anxiety during aging. There was a remarkably conserved activation of the paraventricular nucleus of the hypothalamus, implicated in stress regulation, by MPEP at all investigated ages, whereas the extended amygdala was specifically activated in adulthood only.

**Discussion:**

Our data suggest that neurochemical abnormalities impinging the glutamatergic and GABAergic systems may be responsible for the behavioral phenotype associated with mGlu5 KO animals and point to the close interaction of these molecular players for the development of neuropsychiatric disorders such as schizophrenia. These data could contribute to a better understanding of the involvement of mGlu5 alterations in the molecular imbalance between excitation and inhibition underlying the emergence of a schizophrenic-like phenotype and to understand the potential of mGlu5 modulators in reversing the deficits characterizing the schizophrenic pathology.

